# Investigation of Functionally Graded Adherents on Failure of Socket Joint of FRP Composite Tubes

**DOI:** 10.3390/ma14216365

**Published:** 2021-10-24

**Authors:** Chander Prakash, Vidyapati Kumar, Ankita Mistri, Amrinder Singh Uppal, Atul Babbar, Bhargav Prajwal Pathri, Jonty Mago, Ankit Sharma, Sunpreet Singh, Linda Yongling Wu, Hongyu Zheng

**Affiliations:** 1 Department of Mechanical Engineering, Shandong University of Technology, Zibo 255000, China; zhenghongyu@sdut.edu.cn; 2 School of Mechanical Engineering, Lovely Professional University, Phagwara 144001, India; 3 Department of Mechanical Engineering, Indian Institute of Technology, Kharagpur 721302, India; vidyapatikumar.me@gmail.com; 4 CSIR-Central Mechanical Engineering Research Institute, Durgapur 713209, India; ankitamistri93@gmail.com; 5 Merla Wellhead Solution, 51 Esplanade Blvd Suite 600, Houston, TX 77060, USA; asuppal87@gmail.com; 6 Department of Mechanical Engineering, Guru Gobind Singh Tricentenary University, Gurugram 122505, India; atulbabbar123@gmail.com; 7 Mechanical Engineering Department, JK Lakshmipat University, Jaipur 302026, India; bhargav.pathri@jklu.edu.in; 8 Mechanical Engineering Department, Thapar Institute of Engineering and Technology, Patiala 147004, India; jontymago@gmail.com; 9 Chitkara College of Applied Engineering, Chitkara University, Rajpura 140401, India; ankitsharma.kkr@gmail.com; 10 Department of Mechanical Engineering, Chandigarh University, Gharuan 140413, India; snprt.singh@gmail.com

**Keywords:** bonded joint, tubular socket joint, FEM, FRP composite, functionally graded adhesive

## Abstract

Fiber-reinforced polymer (FRP) matrix materials are quickly being investigated for application in concrete construction repair, reinforcement, and refurbishment. The technology has progressed to the point that its future acceptance is mainly reliant on the availability of established design guidelines based on recognized performance criteria, as well as the cost competitiveness of these technologies in contrast to conventional rehabilitation methods. The goal of this study is to evaluate the different functional grades of adhesives throughout bond length for bonded socket joints of laminated FRP composite pipes. Damage development resistance is high with a functionally graded FRP composite socket joint, as shown. To extend the service life of the structure, the joint designer should use an FRP composite socket joint with a functionally graded adhesive (FGA).

## 1. Introduction

Adhesives are a common option for connecting various materials, especially composites and metals, in a number of industries due to their enhanced durability and strength. When joining composite materials, adhesively bonded joints are preferable since they do not need holes, decreasing stress accumulation across the holes. Adhesively glued composite tube junctions are extensively utilized to couple different composite structures in the aviation and energy sectors. Their low cost of production and high mechanical efficiency structures made of pipes are crucial in the energy and construction industries. Furthermore, as compared to mechanical fasteners, adhesive bonding is usually corrosion-free. Adhesive bonded socket couplings, butt-and-strap connections, heat-activated coupling joints, and flanged joints are the most common joining methods for composite tubes [1]. Attaching composite flanges to pipes typically involves the first three permanent connection techniques. The fundamental architecture of all permanent couplings is the same: two pipe lengths to be connected, a coupler to transmit load to the junction, and a mechanism to transmit force through the tube to the coupler. An overview of bonded pipe connections may assist you in comprehending the essential features of all three kinds of bonded pipe connections.

The first study on tube couplings with adhesive was performed by Lubkin and Reissner [2], who investigated the strength variation of the surface of the adhesive in tube lap joints consisting of thin-walled annular tubular shell sections exposed to axi-symmetric exterior pressure. Volkersen [3] investigated twisting stress in tube lap joints using a fracture mechanics method. Furthermore, Adams and Pepiatt [4] enhanced Volkersen’s [3] assessment by monitoring for adhesives film thickness, and the outcomes of finite element approaches were examined to Lubkin and Reissner’s [2] discoveries. Furthermore, the impact of adhesives fillet and partially taper of adhesive layer on stress concentrations in the adhesive was studied. Chon [5] utilized a mathematical model to investigate the effects of wrap angles, overlap distance, and bonded layer thickness on stress accumulation and especially at the ending of a tube lap junction of FRP composites undergoing twisting. Zhao and Pang [6] devised an empirical framework focused on the mechanics of composite components to examine the impact of composite laminates tubes undergoing twisting and used the maximum strain failure criteria in their findings to anticipate the fracture of glued composite structures. Thomsen [7] investigated the stress concentrations across the epoxy matrix at an adhesion glued lap interface of two orthotropic concentric tubular laminating shells using elasto-static and non-linear assessment. According to Pugno and Carpinteri’s [8] stress characterization of tube glued attachments during loading, maximal shear stresses are produced at the extremities of the adhesives, with the pinnacle of highest shear stress arising at the extremity of the stronger tube. Dragoni and Goglio [9] conducted a comprehensive study on axial stressed tube bonded joints, analyzing and highlighting limitations. The requirement for an empirical, closed-form formulation was also emphasized, and the associated problems were not addressed properly. Using finite element findings on a variety of joint arrangements as mathematical milestones and expanding the investigations given in the literary works were also explored. Goglio and Paolino [10] re-examined Lubkin and Reissner’s [2] classic model for the tube junction under axial stress and developed a Laplace transform-based solution technique. Esmaeel and Taheri [11] investigated the impacts of erosion on the structural behavior of glued tube joints made of composite and aluminum adhesive layer, including the effects of two geometrical and loading parameters on the peel and shear stress variations in the epoxy matrix. Cognard et al. [12] worked on optimizing the joint’s maximal transferred force by evaluating stress distributions in tubular connections confined to lateral loading under the linear elastic hypothesis. Using the small strain-large displacement theory, Apalak et al. [13] examined the geometric non-linear thermally stresses of a tube lap connection under thermal stress on the exterior and interior edges of the pipes as well as different tubular edges situations. Furthermore, when various end conditions were given to the ends of its outer and inner tubes, the results revealed substantial heat strains in the adhesive layer and tubes. In addition, there was substantial deformation in the joint region and high-stress concentration at the adhesive-free ends. Under torsional load, Zou and Taheri [14] investigated stress on adhesively attached socket joints. The socket joints under investigation were made of steel–steel, aluminum–aluminum, and composite–composite adherent combinations. According to this study, combining composite-composite adhesive material substantially enhances the rate of change of shear stress on both the left and right ends of the socket joint. The effect of changing the adhesive shear stress in the socket joint framework on the adherend thickness was examined. Using a finite element analysis simulation technique, Das and Pradhan [15] examined the start and progression of delamination destruction in laminated FRP composite glued tube single lap joints. The Tsai-Wu failure criterion were used to conduct a finite element study of glued tube socket connections of laminated FRP composite materials, and the efficient and safe coupling distance for satisfactory joint quality was discovered. Stresses were observed to be localized at the unrestricted boundaries and adherend junctions in the socket joint coupling zone, where they were susceptible to three-dimensional impacts. Das [16] investigated the use of FGA rather than laminated FRP composite tube adherends in a bonded tubular single lap joint (TSLJ) employed to uniaxial tensile loading using the ANSYS Parametric Design Language (APDL), with the main goal of lowering the accumulation of stress in the joint area. By varying the flexibility of each layer in the thickness direction, Dos Reis et al. [17] explored the layered FGA. Different stiffness variation ratios and adhesive characteristics for single lap joints were also quantitatively evaluated using cohesive zone modeling, comparing the stress variation of the layer of adhesives and the resulting joint strength. In addition, an optimization technique for typical graded material attributes is presented, which takes alternate distribution laws such material weight and strength into account. Redmann et al. [18] examined the edge-bending moment for the two joint designs using conventional mathematical models [1,2]. Experiments were carried out to assess these investigations, as well as the reasons for each design’s failure. Finite element analysis was also utilized to investigate stress distributions along the bondline, and experimental testing were carried out to assess these studies as well as the reasons for each design’s failure.

According to the previous literature review, elasticity mismatches or peel strength in all kinds of tube junctions produce stress peaks or stress anomalies at the bond line’s borders in nearly all bonded joints. Relief grooves [19,20,21], scarf joints [22,23], and rounding edges [22,23] have all been suggested in the literature as methods to decrease the degree of uniqueness of these peaks. Although these methods may be used for testing, they are more difficult to use in a commercial setting. Recent nanotechnology advancements and applications in adhesives have resulted in significant improvements in the mechanical properties of polymers and joints [24]. The elastic modulus may be adjusted constantly by varying the amount of reinforcement in FGA [25,26,27]. Moreover, many previous studies have shown an interest in investigating the machining capabilities of FRP composites utilizing unconventional machining techniques [28,29,30,31,32].

The problem would be exacerbated by tube connections made of laminated FRP composites and FGA materials. Peak adhesive stresses have been found to be reduced using blended adhesives and FGA. Mixed adhesives, also known as dual adhesives, comprised of two types of adhesives designed to minimize stress accumulation in brittle adhesive joints by adding a flexible adhesive at the overlap’s ends. In recent years, FGA—which has a clean and consistent shift in the adhesive’s mechanical characteristics—has received a lot of attention. In this regard, mixed adhesives are FGA with a stepwise functional grading. Because it is simple to manufacture and utilizes shear loading, the most effective form of adhesive loading—the lap joint—is the most frequent type of joint in the business. Because of its simplicity and effectiveness, the single lap joint (SLJ) with metallic or composite adherents is the well-researched joint in the literature and the most often observed in industrial application. The existence of stress accumulation towards the edge of the adhesive layer, on the other hand, is a significant fault in these connections. Early joint failure occurs at the overlap’s ends when the adhesive is brittle or low-transverse-strength composites or adherents are used. As a consequence, discovering methods to decrease these stress concentrations in order to enhance adhesive joint strength is one of the most significant areas of study in the field of adhesive bonding.

The sequence in which the rest of the article is structured is as follows: The modeling of a tubular socket joint with meshing properties is covered in Section 2. Section 3 shows the results of different functional adhesive grades for bonded socket joints of laminated FRP composite pipe throughout the bond length followed by Section 4 which presents the conclusion.

## 2. Modeling of Tube Socket Joint

Theoretical mathematical modeling can almost always replace costly scientific experiments while also giving additional insight than can be gained physically. Theoretical mathematical modeling has already played an important role in Functionally graded modified (FGM) research, and it will continue to play an essential role in future advancements owing to the significant complexity involved. The finite element method (FEM) is a numerical approach used to conduct finite element (FE) analysis of any given physical event, which aids in fully understanding and quantifying any physical occurrence. 3D geometrically FE calculations are utilized in this study to predict the development of pre-existing failure at the interface in a tube socket joint under axial stress. A variety of simulations were run to investigate the possible impact of FGM on the structural integrity of the joint.

### 2.1. Constrained Boundary Condition

**Figure 1:**
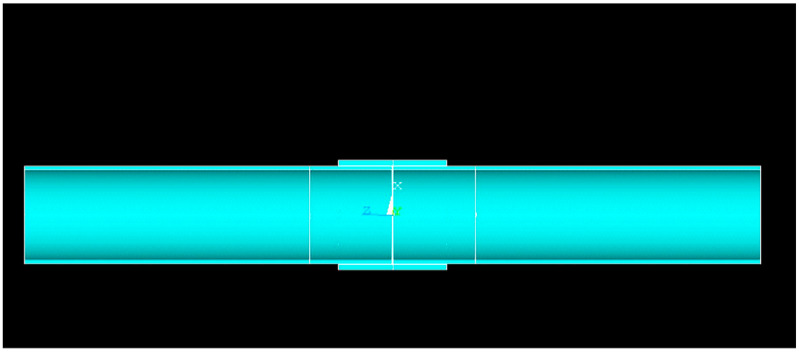
Discretized cylindrical socket joint layout.

Simulating cylindrical joints under concentrated loading is accomplished by fixing one end of the tube and applying an extensible stress of 10 MPa to the other. The restricted boundary condition used in this FE simulations for a tube sockets connection bearing axial force is shown in Figure 1.

For Z = –L; U = V = W = 0

Where,

2L = The structure’s total overall length

Z = distance along the combined structure

W, V, U = longitudinal, circumferential, radially displacement associated with cylindrical co-ordinate system (z-θ-r), respectively.

### 2.2. Meshing Detail-Oriented

In this research, 3D block components of tube socket connection are used in FE analysis. SOLID 46 iso-parametric, three-dimensional eight nodal layered volumetric components were utilized to simulate the tubes made of composite materials and socket coated layer-by-layer in this FE study, with orthotropic material characteristics considered for each ply. SOLID 45 bricks were used to represent the isotropic adhesive layer. There are eight nodes in each of these elements, each with three translational degrees of freedom. The sticky layer is the element size in the FE model (0.1 mm, 0.2530 mm, 0.05 mm). This research used a mesh shaped arrangement with 360 components present across the orientation of circumference, 260 elements present across the direction of axis, and two elements present along the direction of radius in order to appropriately discretize the layers of adhesive. Figure 1 depicts a cylindrical socket joint construction with several mesh densities used during the discretization process, which replaces the continuum with a finite collection of points. As a result, it is critical to investigate the damage behavior of functionally modified adhesives on failures of socket attachment joints of laminated fiber reinforced polymer composites using ANSYS software (version 18.1) in order to discover a reliable FGA material that can be effectively integrated with FRP composite and made available for industrial use. Table 1 illustrates the geometrical characteristics of a bonded cylindrical socket. Furthermore, Table 2 shows the layer-by-layer material characteristics for orthotropic tubes/sockets, whereas Table 3 exhibits the epoxy resin’s elastic properties [33,34]. In this study, the meshing technique proposed by Nimje and Panigrahi [34] was used to simulate the tubular socket joint.

**Figure 2:**
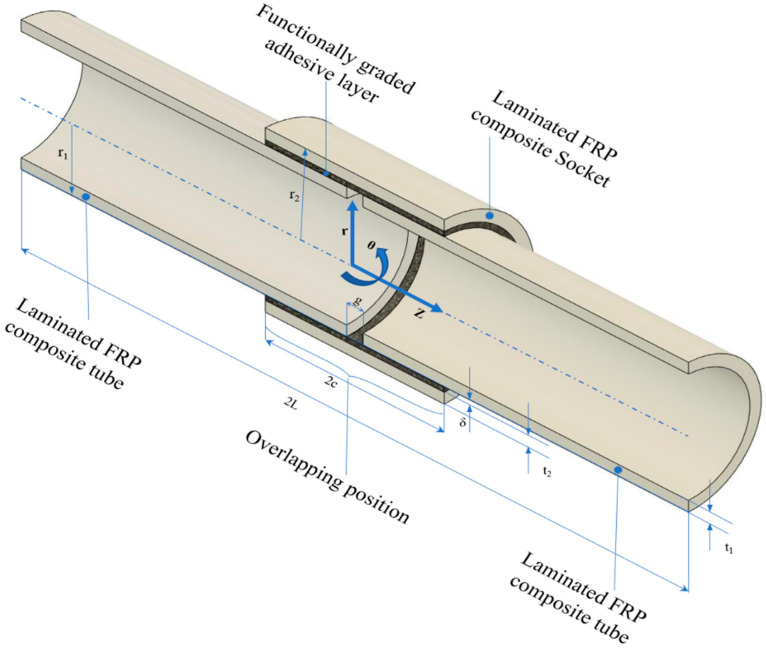
Geometrical design of FGA socket joint.

Figure 2 depicts the geometrical design of the FGA socket joint. It can be observed that two laminated FRP composite tubes have been put close together to create a connection with radius r1 and thickness t1. The FGA layers of thickness δ are then used to fill the space between the two laminated FRP tubes. A laminated FRP composite socket with an overlapping section of distance 2c is put over it.

## 3. Results and Discussions

### 3.1. Case I

The Equations (1)–(4) reflect the different functional gradings for adhesives throughout bond-distance for bonded socket joints of coated FRP composite pipes. In this context, it is desirable to introduce a ratio parameter known as fixed modulus ratio (R), which is a ratio of the highest value of elasticity modulus and the lowest value of elastic modulus of adhesive. For the constant modulus ratio R = 2, the maximum modulus E_m_ is 2800 MPa, while the lowest modulus E_o_ is 1400 MPa. These roles are compared to the modulus ratio of an isotropic adhesive, R = 1 (mono-modulus adhesive).

The following are the various sorts of functional assumptions [34]

(1) $$E_{1}\left(z\right)=E_{m}-4\left(\frac{E_{m}-E_{o}}{c^{2}}\right)z^{2}$$

(2) $$E_{2}\left(z\right)=E_{m}-16\left(\frac{E_{m}-E_{o}}{c^{4}}\right)z^{4}$$

(3) $$E_{3}\left(z\right)=E_{m}-\frac{E_{m}-E_{0}}{L^{2}}{\left(z-c\right)}^{2}$$

(4) $$E_{4}\left(z\right)=E_{o}+\frac{E_{m}-E_{0}}{L^{2}}{\left(z+c\right)}^{2}$$

The max and min elasticity moduli of the glue (adhesive) are E_m_ and E_o_ respectively. The bonded joint region’s coupling distance (2c), and the length z is computed throughout the bond length L. Smooth changes of bond layer modulus have previously been achieved [27] by applying a number of rings of adhesive with varying moduli in the bond line, which is represented by the linear function profile in Equations (5) and (6).

(5) $$E\left(z\right)=E_{o}+\frac{E_{m}-E_{o}}{\pm C/2}z$$

(6) $$E\left(z\right)=\frac{E_{o}-E_{m}}{\pm C/2}z+2E_{m}-E_{o}$$

Failure at the socket junction—i.e., adherence inability at the pipe-adhesive contact—will be investigated using the Tsai-Wu criterion. The joint’s crucial zone is located at the borders and towards the centre of the glued tube socket joint, according to the failure indices of the socket joint.

Asymmetrical and symmetrical functions will be considered as two forms of functional grading. Equations (1)–(4) may be used to change the various functional grades, the failure indices will be determined.

#### 3.1.1. Gradation by Symmetric Functions

**Figure 3:**
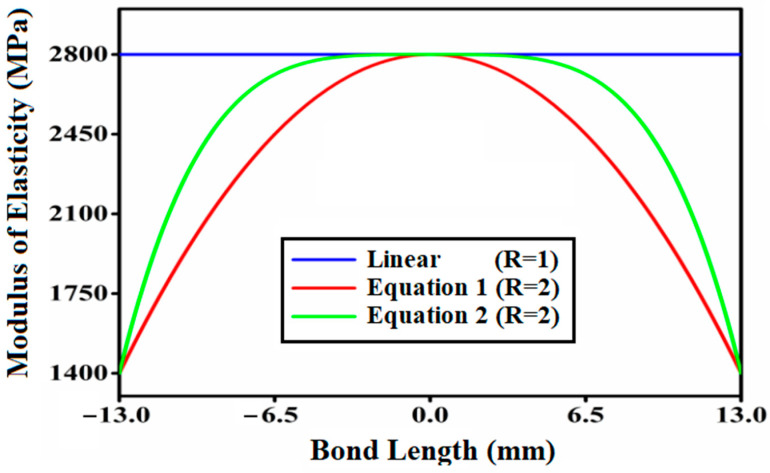
Parabolic fluctuation of modulus of elasticity (E) of the adhesive layer with modulus ratio R = 2 (Equations (1) and (2)).

The variability of elastic modulus with bond length for symmetric function profiles with modulus ratio 2 and adhesive with modulus ratio 1 is depicted in Figure 3. It is worth noting in this context that the figures were acquired utilizing the Origin 2019b application. Figure 3 depicts the parabolic variation of the adhesive layer’s modulus of elasticity (E) with three different modulus ratios R = 1, 2, 3. The bonded tubular socket joint illustrated in Figure 3 has the lowest modulus of elasticity (1400 MPa) at the extremities and the highest modulus of elasticity (2800 MPa) in the center.

**Figure 4:**
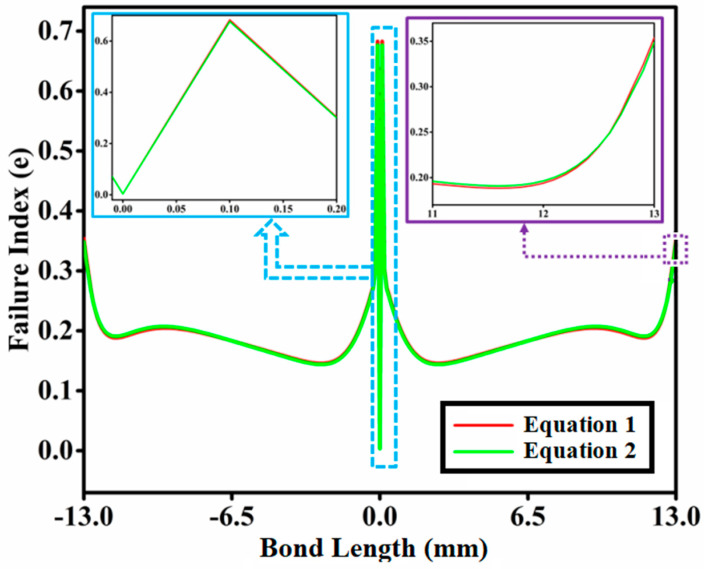
Variation of the failure index (e) along the length of the bond (Equations (1) and (2)).

**Figure 5:**
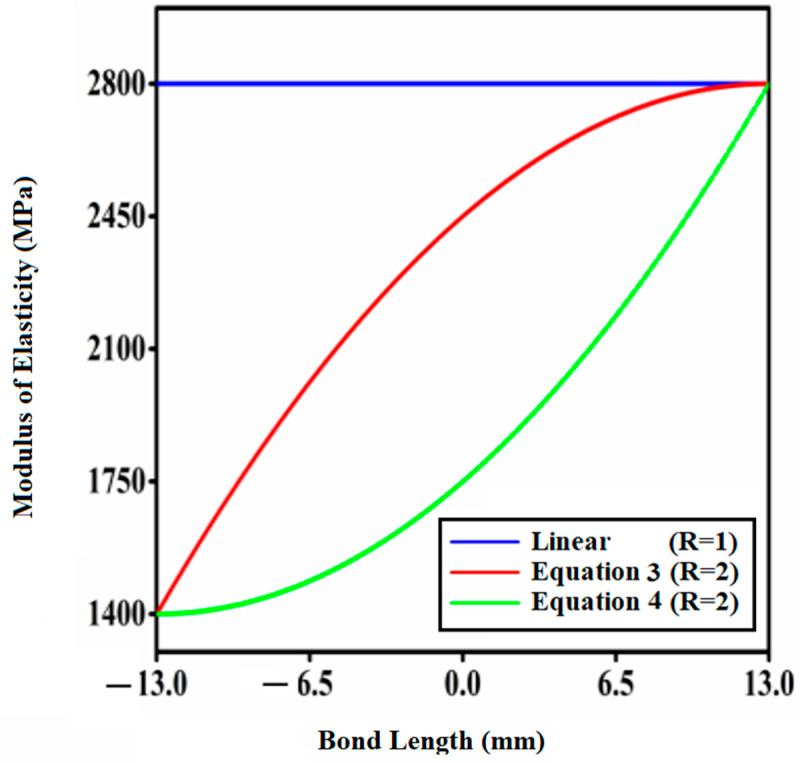
Asymmetric fluctuation of the adhesive thickness’s elastic modulus (E) along the modulus ratio R = 2 (Equations (3) and (4)).

**Figure 6:**
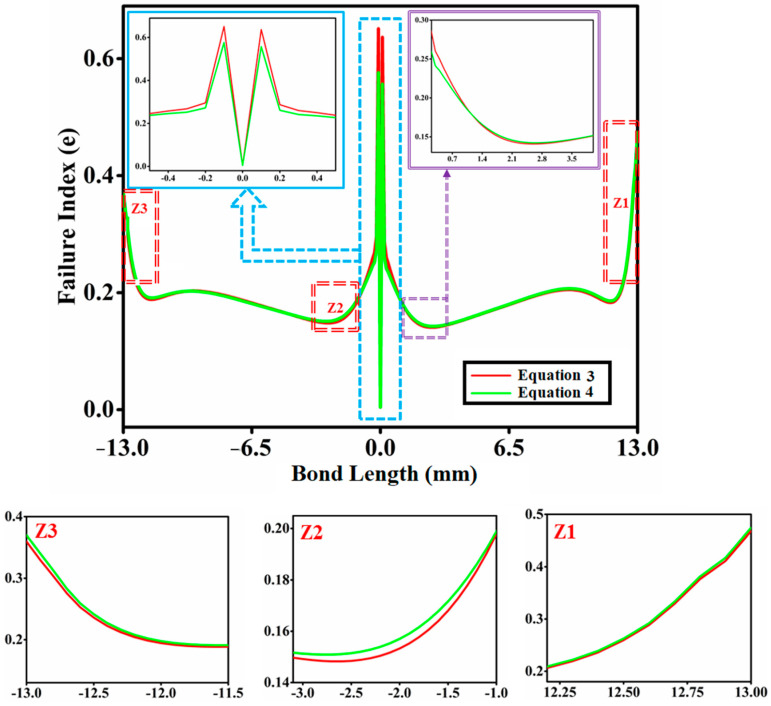
Fluctuation in the fatigue index (e) of asymmetric fluctuation throughout the bond length (Equations (3) and (4)).

The failure index (e) is the parameter used to assess whether or not the bonded tubular socket joint assembly is prone to failures. Tubular socket joints will fail if the failure index (e) is greater than or equal to one, otherwise, there will be no failure. Figure 4 depicts the distributions of failure indices throughout the bond length of the interfacial surface of a tubular socket joint with functionally graded adhesive using Equations (1) and (2). The failure index (e) along the bond length is calculated using the Tsai-Wu failure criterion and shown in Figure 4. The failure index for Equation (1) is somewhat higher at the joint edges of the joint, with a peak around the middle of the bonded tubular socket joint. At bond lengths of ±12.6 mm, ±6.5 mm, and 0 mm, the failure index value is identical. Figure 4 shows that when a functionally graded bond layer is employed, there is a substantial decrease in peak values of failure indices at the free edges of both tubes’ adhesive interfaces in the coupling area. Similarly, Figure 5 depicts the asymmetric fluctuation of the adhesive thickness’s elastic modulus (E) along the modulus ratio R = 2 using Equations (3) and (4), and Figure 6 depicts the variation in the fatigue index (e) across the bond length using Equations (3) and (4). Figure 6 shows the asymmetric variation in the fatigue index (e) throughout the bond length of Equations (3) and (4). Figure 6 demonstrates that when a functionally graded bond layer is used, the peak values of failure indices at the free edges of both tubes’ adhesive interfaces in the coupling region, denoted as Z1 and Z3, drop significantly.

#### 3.1.2. Effect of the FGM on the FRP Composite

The purpose of this study was to see how functionally modified adhesive affected attachment joint failures in laminated FRP tubes. The failure study of tube and socket joints of various functional modified adhesives with mounting modulus quantitative relationship has been carried out in this research. The uneven FGM adhesive with Equation (4) has a higher failure resistance property than the FGM adhesive with Equation (3) under axial stress circumstances. The uneven FGM adhesive showed a higher failure growth resistance in the center of the junction when compared to the symmetric FGM adhesive. As a result, recommendations are frequently made for the use of FGM adhesive that has been altered to perform unevenly.

The four distinct forms of functional grading, such as power law function grading, are contrasted. Changing the functional grade according to Equations (1)–(4) will determine the failure indices.

### 3.2. Case II

#### Gradation by Even Power Law Functions

The following are the modulus parameters in normalized form for the FGM adhesive cylindrical junction, as well as the profiles that were considered [34].

(7) $$E_{f1}^{n}=\frac{E_{f1}}{E_{m}}=\exp\left[-4\ln\left(\frac{E_{m}}{E_{0}}\right){\left(\frac{z}{2c}-\frac{1}{2}\right)}^{2}\right]$$

(8) $$E_{f2}^{n}=\frac{E_{f2}}{E_{m}}=4\frac{E_{0}-E_{m}}{E_{m}}\left[{\left(\frac{z}{2c}\right)}^{2}-\left(\frac{z}{2c}\right)\right]+\left(\frac{E_{m}}{E_{0}}\right)$$

(9) $$E_{f3}^{n}=\frac{E_{f3}}{E_{m}}=8\frac{E_{m}-E_{0}}{E_{m}}\left[2{\left(\frac{z}{2c}-\frac{1}{2}\right)}^{4}-{\left(\frac{z}{2c}-\frac{1}{2}\right)}^{2}\right]+1$$

(10) $$E_{f4}^{n}=\frac{E_{f4}}{E_{m}}=\frac{64}{5}\frac{E_{m}-E_{0}}{E_{m}}\left[{\left(\frac{z}{2c}-\frac{1}{2}\right)}^{6}+{\left(\frac{z}{2c}-\frac{1}{2}\right)}^{4}\right]+1$$

The many forms of functional grading, such as power law gradation, will be considered. Changing the different functional grades according to Equations (7)–(10) will determine the failure indices.

**Figure 7:**
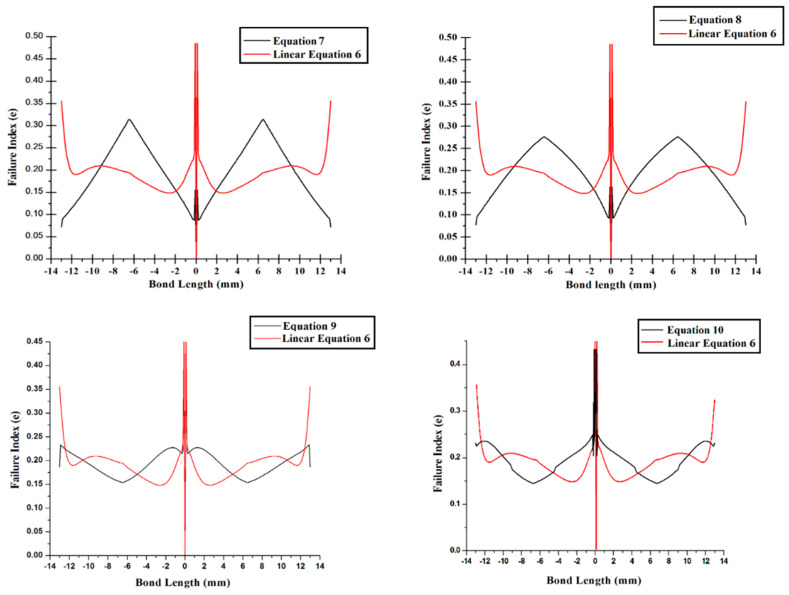
Variation in the breakdown index throughout the bond length for Equations (7)–(10).

Tsai-Wu failure criterion findings vary throughout the bond length illustrated in Figure 7 due to the several even power law expressions provided by Equations (7)–(10).

In the middle of the junction, the breakdown index for Equation (7) of the linear equation is smaller than for Equation (6), and the stress peak is lower on the side of the joint than for Equation (6) of the linear equation, as shown in Figure 7. The failure index value is zero in the center of the socket joint, but it is about 0.30 at the bond lengths of 6 mm and −6 mm.

For each collection of nodes on the adhesive’s internal surface, hoop and radial stresses have been estimated, which are primarily accountable for breakdowns under circumferential pressure loading. The stress results are expected to be uniform for all values of angles with respect to the circumferential axis (θ). As a result, the hoop and radial stress for various interweaving over these nodes have been determined. It shows how the peak values of stresses at the pipeline’s edges converge when the first section of the seamlessly blended pattern is along the radial direction.

Furthermore, when compared to Equation (6) of the linear equation, the breakdown index for Equation (9) has a lower peak along the side of the joint, and at bond lengths 13 mm and −13 mm, it is strictly rising to Equation (6). The failure index for Equation (10) is similarly lower towards the junction ends and peaks in the center of the bonded tubular socket joint, as demonstrated in Figure 7. At bond lengths of ±12.6 mm, ±6.5 mm, and 0 mm, the failure index value is identical.

### 3.3. Effect of the FGM on the FRP Composite

It is critical to enhance the fracture/bond failure characteristics of the material to increase the strength and serviceability of the pipe socket joint in various industrial applications such as underground mine water drainage through pipe to a reservoir, etc. The impact of FGA on the interfacial failure transmission rate is demonstrated at the crucial site of the glued tube socket junction, which arises from the edge of the tube surfaces’ coupled zone in tube socket joint. Resistance to high-failure growth reduces propagation and increases the life of tubular socket joints.

**Figure 8:**
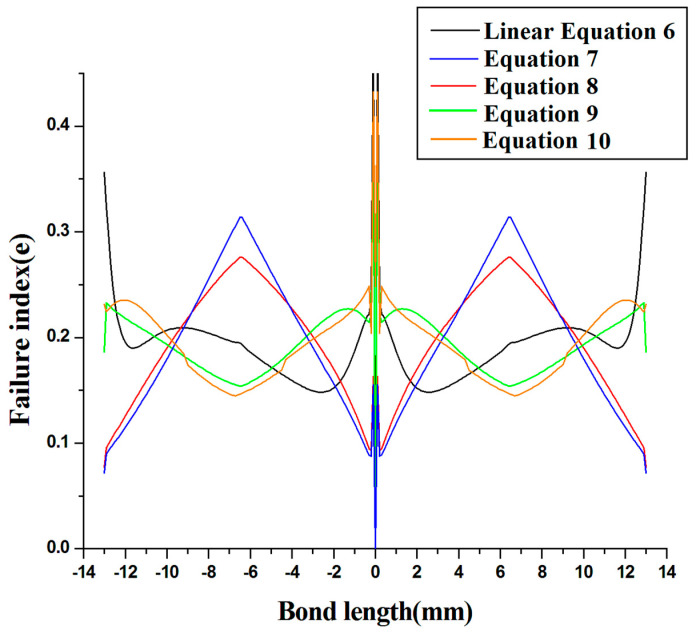
Failure indexes of various even power law profiles compared to linear profiles.

As a result, the use of an FGA layer in the tube socket connection can be recommended. As a result, it is critical to compare all profiles in a single plot. Figure 8 compares the different even power laws of Equations (7)–(10) to the linear Equation (6) to determine which equation is the best for lowering the stress intensity factor at the side of the joint and in the center. In this context, the stress intensity factor is defined as the size of the stress singularity, which is utilized in fracture mechanics to anticipate the stress state near the tip of a crack or notch produced by a distant load or residual stresses.

The Equation (9) findings are compared to the linear Equation (6) graded FGA. When an even power profile FGA modulus-ratio (R) layer ranging from 2 is utilized, significant reductions in top failure indices of up to 25–55 percent are observed close to the unbound edges and in the center of the pipe joint, as well as in the bonding zone, of both tubes–adhesive interface.

## 4. Conclusions

Fiber reinforced polymer matrix materials are rapidly being researched for use in a variety of manufacturing and service sectors, and the technology has advanced to the point where its future acceptance is primarily dependent on the availability of established design guidelines based on recognized performance criteria. In view of the current demand, it is critical to address the reliable FGA material that can be effectively integrated with FRP composite and made available for industrial usage. In this paper, damage study of functionally modified adhesives on failures of socket attachment joints of laminated fiber reinforced polymer composites was conducted to determine critical damage start locations using ANSYS software. Furthermore, the failure analysis of different functional modified adhesive tube and socket joints with mounted modulus quantitative relationship has been addressed. The following specific conclusions were made based on the findings and observations about the damage in the FRP composite socket joint:

1. Case I investigates the impact of FGA on socket junctures failures in laminated FRP tube. The investigation of failure of tube and socket junctures with varied functional grading adhesives and fixed modulus ratios was carried out in this work. In Case I, under vertical stress conditions, the uneven FGM adhesive with Equation (4) was found to have superior failure resistance qualities than the symmetric FGM adhesive with Equation (3). When compared to FGM adhesives with symmetric functions, the asymmetric FGM adhesive was shown to have greater failure growth resistance in the middle of the joint in Case I. As a result, in Case I, using FGM adhesive with an asymmetric function is recommended since it produces superior outcomes.
2. In Case II, several profiles of even power law are compared to the linear Equation (6), and it is determined that Equation (9) is a superior functional graded adhesive joint because it reduces stress concentration at the side joints, with a failure index of 0.25 at bond lengths of −13 mm and 13 mm. The stress accumulation at the juncture’s center was found to be less, up to 0.3, as compared to the linear profile Equation (6) and distinct even power law Equations (7)–(10). Although this may be ignored, it has also been discovered that the components of the FGM graded by Equation (9) will lessen stress accumulation at the side and center of the joint.

As a result, it can be revealed that damage growth resistance is excellent with a functionally graded FRP composite socket joint, and the joint designer is recommended to install an FRP composite socket joint with an FGA to prolong the serviceability of the structure.

## Figures and Tables

**Table 1:** Geometrical characteristics of bonded cylindrical socket.

Parameters of Tube and Socket	Gr/E-Coated FRP (T300/934)
Arrangement of plies	[0/90]S [33]
Thickness of the adhesives	δ = 0.1 mm [33]
The structure’s total length	2L = 178 mm [34]
Tube’s outside dimension	r_1_ = 14.4 mm [34]
Socket’s outside radius	r_2_ = 16 mm [34]
Thickness of tube	t_1_ = 1 mm [34]
Thickness of the coupler	t_2_ = 1.5 mm [33]
Length of coupling	2c = 26 mm [33]
Space among the tubes	g = 0.2 mm [33]

**Table 2:** Layer by layer material characteristics for orthotropic tube/socket.

**Material Characteristics**	**Values**
Young modulus along axial E (z) axis	127.50 GPa [34]
Young modulus along radial E (r) axis	4.80 GPa [27]
Young modulus along radial E (θ) axis	9.00 GPa [33]
Modulus of rigidity along two axis is G_zr_ = G_z__θ_	4.80 GPa [33]
Modulus of rigidity along G_θ__r_	2.55 GPa [27]
Poisson’s ratio along two axis is µ_zr_ = µ_z__θ_	0.28 [33]
Poisson’s ratio along two axis is µ_θ__r_	0.41 [33]
**Strengths**	**Values**
Transverse strength $R_{T}$	49 MPa [27]
Shear strength	2.55 MPa [27]

**Table 3:** Epoxy resins elastic characteristics.

Elastic Properties of Resin	
**Epoxy resin’s elasticity (E)** **Poisson’s ratio (µ)**	2.8 GPa [33]
	0.4 [27]
**STRENGTH OF RESIN**	
Yt	65 MPa [33]
Yc	84.5 MPa [27]

## Data Availability

The data presented in this study are available on request from the corresponding author.
